# Looking for Age Differences in Self-Driving Vehicles: Examining the Effects of Automation Reliability, Driving Risk, and Physical Impairment on Trust

**DOI:** 10.3389/fpsyg.2019.00800

**Published:** 2019-04-26

**Authors:** Ericka Rovira, Anne Collins McLaughlin, Richard Pak, Luke High

**Affiliations:** ^1^Department of Behavioral Sciences and Leadership, US Military Academy, West Point, NY, United States; ^2^Department of Psychology, North Carolina State University, Raleigh, NC, United States; ^3^Department of Psychology, Clemson University, Clemson, SC, United States

**Keywords:** autonomous cars, self-driving vehicles, older adults, cognitive aging, automation reliability, individual differences, trust, technology adoption

## Abstract

**Purpose:**

Self-driving cars are an extremely high level of autonomous technology and represent a promising technology that may help older adults safely maintain independence. However, human behavior with automation is complex and not straightforward (Parasuraman and Riley, 1997; Parasuraman, 2000; Rovira et al., 2007; Parasuraman and Wickens, 2008; Parasuraman and Manzey, 2010; Parasuraman et al., 2012). In addition, because no fully self-driving vehicles are yet available to the public, most research has been limited to subjective survey-based assessments that depend on the respondents’ limited knowledge based on second-hand reports and do not reflect the complex situational and dispositional factors known to affect trust and technology adoption.

**Methods:**

To address these issues, the current study examined the specific factors that affect younger and older adults’ trust in self-driving vehicles.

**Results:**

The results showed that trust in self-driving vehicles depended on multiple interacting variables, such as the age of the respondent, risk during travel, impairment level of the hypothesized driver, and whether the self-driving car was reliable.

**Conclusion:**

The primary contribution of this work is that, contrary to existing opinion surveys which suggest broad distrust in self-driving cars, the ratings of trust in self-driving cars varied with situational characteristics (reliability, driver impairment, risk level). Specifically, individuals reported less trust in the self-driving car when there was a failure with the car technology; and more trust in the technology in a low risk driving situation with an unimpaired driver when the automation was unreliable.

## The Importance of Driving for Older Adults’ Well-Being

By 2030, the proportion of the U.S. population aged 65 and older is expected to double to about 71 million older adults, or one in every five Americans (Federal Interagency Forum on Aging Related Statistics, 2008). Like today, the vast majority of these future older adults will want to maintain independence (Willis, 1996); living at home and not in an assisted or independent facility. A common reason for an older adult to move to an assisted living facility is because they, due in part to age-related changes in cognition (Parasuraman and Nestor, 1991; Salthouse, 1996; Tracy and DeYoung, 2004), can no longer carry out everyday activities such as driving.

Driving is the most frequent mode of transport for those above age 65 (Jette and Branch, 1992; Rosenbloom and Waldorf, 2001). Independent mobility is also a major component of older adults’ sense of functional independence (Dellinger et al., 2001; Adler and Rottunda, 2006) even when alternative public transportation is available (Adler and Rottunda, 2006). So crucial is the sense of independence from driving that driving cessation is associated with decreased out-of-home activities (Marottoli et al., 2000; Huisingh et al., 2016), increased depressive symptoms (Fonda et al., 2001), and contributes to a variety of health problems (Chihuri et al., 2016).

Literature in automation, aging, and transportation has shown mixed findings regarding older adults’ adoption of assistive or automated driving technology. While some mild and common forms of automation, such as automatic transmission, enhance older adults’ driving performance and are readily accepted (Selander et al., 2011), current opinion surveys suggest strong distrust of higher degrees of autonomous technology in older adults (Schoettle and Sivak, 2015; Becker and Axhausen, 2017; Abraham et al., 2018; Hulse et al., 2018). However, initial strong distrust should not be taken to mean that older adults will not adopt self-driving cars. Contrary to the generally accepted stereotype of older adults not using or wanting new technology, older adults’ attitudes toward technology are quite open and positive (Rogers et al., 1996; Czaja and Sharit, 1998). Older adults will readily adopt technology when explicit benefits are understood (Melenhorst et al., 2006). The barriers to actual adoption are related to poor usability, access (e.g., the cost is high), or a lack of understanding of the cost/benefits of adopting that technology (Melenhorst et al., 2001). Nonetheless, the results of recent surveys have suggested that trust in self-driving technology is extremely low in the general population but especially older adults suggesting that older adults do not yet perceive the benefits or question the usability. It is with this background that the current research is focused on understanding the situational, individual, and technological conditions under which older adults will trust self-driving vehicles compared to the judgments of younger adults.

### Automation and Trust

In general, older adults tend to suffer the negative performance effects of imperfect automation more than younger age groups. More specifically, they tend to over-depend and over-trust automation (Fox and Boehm-Davis, 1998; Ho et al., 2005b; Donmez et al., 2008; Pak et al., 2012, 2014). Where trust is defined as an attitude (Lee and See, 2004) and reliance is defined as overt behavioral dependance. This over-trust and over-reliance may come from older adults’ inability to properly identify and diagnose automation errors due to age related limitations in working memory (Pak et al., 2016a). Lower working memory may also inhibit older adults’ ability to properly calibrate their trust by making it more difficult to integrate previous instances of unreliable automation sessions into a coherent and up-to-date mental model (Sheridan, 1992; Gilbert and Rogers, 1999; Ho et al., 2005a). Lower working memory may also make it more difficult for older adults to generate alternative courses of action, a working memory-intensive activity, if they are conscious of an automation failure (Parasuraman et al., 2000). Adding further potential complexity, more recent research has shown that individual differences and age induces different responses to different degrees of autonomy (Pak et al., 2016a; Rovira et al., 2016). Thus, it is important to include age in studies of automation trust and use.

In contrast to experimental results that show older adults’ over-trust of automation, in the driving domain, recent opinion surveys of a lifespan sample of adults showed that older adults showed more negative opinions of self-driving cars than younger adults (Schoettle and Sivak, 2015, 2016). For example, in response to the question, “if the only vehicles available were completely self-driving, how concerned would you be about riding in such vehicles?”, 41% of older adults were very concerned compared to only 21% of younger adults. In 2016, when asked about the preferred level of automation (no self-driving to completely self-driving), 56% of older adults preferred no self-driving compared to 41% of younger adults (Schoettle and Sivak, 2016). This negative preference for completely self-driving cars has increased since 2015 (older adults: 50%, younger adults 35%; Schoettle and Sivak, 2015). But these opinion surveys are in direct contrast to experimental results that show older adults having higher trust compared to other age groups for lower forms of vehicular automation (e.g., Donmez et al., 2008; Pak et al., 2016b).

What might explain older adult’s relative distrust with self-driving cars? Given the importance of maintaining mobility, older adults should be more accepting of technologies that help them maintain independence. Indeed, older adults’ high trust of transportation-related automation, even when imperfect, was observed in a study by Donmez et al. (2008). This finding of older adults’ mis-calibrated trust toward transportation-related automation was also found in a study examining trust in four domains of automation that found that older adults trusted transportation automation more than any other domain (e.g., health automation) and more than younger adults (Pak et al., 2016b). A possible explanation for older adults’ differential trust in transportation automation could be their heightened importance of independent mobility, compared to other age groups, and the ramifications of losing it. Thus, the literature is mixed, but shows a level-of-automation effect on trust such that older adults over-trust moderate forms of transportation-related automation (e.g., Donmez et al., 2008) and distrust high forms of transportation-related automation (Schoettle and Sivak, 2015, 2016).

While self-driving cars are a major area of research, no manufacturer currently (as of early 2019) sells a vehicle with full self-driving capabilities to the public. Thus, drivers are left to form opinions of self-driving cars from media reports or their own experiences with lower forms of transportation automation (e.g., adaptive cruise control). Current sentiment of self-driving cars tends to skew on the negative side. This is important because perceptions of reliability of automation is one of the strongest predictors of trust in automation (Lee and Moray, 1992, 1994; Lee and See, 2004; Hancock et al., 2011). A recent study showed that trust is differentially influenced by automation depending on the domain of automation, even when all other factors (e.g., reliability) are controlled (Pak et al., 2016b), urging caution and additional research to examine the factors that affect trust in this new area.

## Overview of the Study

The purpose of the current study was to examine the factors that affect younger and older adults’ perceptions of self-driving vehicles. A nationwide survey found that for older adults, among the choices of alternative transportation, having an alternative volunteer driver (i.e., community residents chauffeuring seniors to their destination) was most preferred over busses or taxis (Rahman et al., 2016). Thus, short of ceasing to drive, self-driving cars may represent the closest analog to the most preferred alternative mode of transportation compared to shuttle busses or taxis. The extant research, most of which is survey-based, has not examined what specific factors lead drivers to distrust self-driving cars. The aforementioned review of the driving cessation literature has shown that for older adults, the ultimate decision to cease driving, and thus be more open to alternative transportation technology, is based on factors related to the *driver* (e.g., normative age-related changes, disability status), the *situational risk* (e.g., driving in bad weather, at night) while the automation literature has shown that the major facilitator to trusting technology (and thus adoption and usage) is its performance or *reliability* (Lee and See, 2004; Hancock et al., 2011).

Research has shown that when users are given more explicit information regarding the limitations of driver assistance systems, their acceptability of those systems changes (Biassoni et al., 2016), suggesting that the distrust exhibited by older adults to self-driving cars may be changeable. However, little research has examined the flexibility of older adults’ trust in vehicle technologies, and the factors that affect it, especially for fully autonomous cars.

Older adults are sensitive to the overall reliability of technology (Mitzner et al., 2010), and thus we expected this awareness to affect their perceptions. Will older adults’ trust in self-driving cars reflect changing reliabilities or variations in risk due to the situation or unique driver circumstances? The current research manipulated three key factors that have been shown to influence both driving cessation and trust in technology: (1) reliability of technology (Hancock et al., 2011), (2) driving risk (Naumann et al., 2011), and (3) perceived level of driver impairment (Ball et al., 1998) to examine their relative independent and interactive influences on older adults’ trust in self-driving vehicles. Although self-driving cars are hypothetical, there is a large body of literature in aging and automation that suggests several hypotheses.

(1) We expected that trust in self-driving cars would be influenced by failure of the self-driving car (reliability). This hypothesis was based on a meta-analysis that found reliability of automation influenced trust (Hancock et al., 2011).
(2) We expected that for older adults, increased situational risk would increase trust in the self-driving car. This hypothesis was based on the driving cessation literature showing that older adults frequently altered their driving behavior and strategies to account for the increased risk in driving due to age-related declines in sensation, perception, and cognition (Naumann et al., 2011). It is also based on the notion that increased situational risk would make benefits of a self-driving car evident to older adults (Melenhorst et al., 2001).
(3) Given the effect of perceived level of driver impairment on driving cessation decisions (Ball et al., 1998), we expected that trust would be higher for self-driving vehicles if the driver appeared impaired compared to no impairment again, because the benefits are made evident that they will be more in need of a self-driving car.
(4) We expected that, for both age groups, situational risk, driver impairment status, and car reliability would interact to affect trust. While reliability should ultimately determine trust, the effect would be moderated by driver impairment status and risk level. We did not have specific hypotheses regarding age differences given the dearth of prior research. However, the direction of the hypotheses was informed by recent work that showed age differences in trust for transportation-related automation (Pak et al., 2016b)

Studying technology that is not yet widely available was challenging as we could not expose participants to actual self-driving cars. Thus, we used a factorial survey, commonly used in the sociological literature when the desire is to assess how independent factors might affect perceptions (Auspurg and Hinz, 2015). The factorial survey assesses subjective perceptions (i.e., trust) after presenting a vignette or scenario that describes the outcome of a driver of a hypothetical self-driving car. This method has also been used in other human factors research (e.g., McLaughlin et al., 2013), including the study of automation (Endsley and Kiris, 1995).

### Methods

#### Participants

*A priori* analyses showed a minimum of 126 participants were required to detect an effect size of 0.2 (power level of 0.8 and alpha at 0.05; Erdfelder et al., 1996). A total of 138 participants were surveyed; 86 younger adults and 52 older adults. The older adults were community-dwelling and independent-living (i.e., did not reside in a care facility). The younger adults were recruited using Amazon’s Mechanical Turk platform (MTURK). MTURK is an online platform powered by Amazon that recruits a large and diverse participant pool, compensating participants based on the difficulty and length of the task, defined as a Human Intelligence Task (HIT), and providing data at least as reliable as data obtained via traditional methods (Buhrmester et al., 2011).

#### Materials

##### Demographic surveys

We gathered biographical data from our participants including technology experience, automation complacency, and life space extent of mobility. Scores on these measures were used to describe our sample.

Technology experience was measured using the short form of the computer proficiency questionnaire (CPQ-12; Boot et al., 2013). The CPQ-12 has been shown to be a reliable (Cronbach’s alpha = 0.95) and valid indicator of computer proficiency especially for older adults. Participants indicated their comfort with six areas of technology (e.g., printing, email) on a 5-point Likert scale. The mean ratings for the six domains was summed to create a total score. Scores could range from 0 to 30 with higher scores indicating greater proficiency.

Pre-existing attitudes toward general automation was measured with the complacency potential rating scale (CPRS; Singh et al., 1993). CPRS is a 20-item questionnaire where participants indicated the extent they agreed with statements about automation on a 5-point Likert scale. Scores could range from 0 to 100 with higher scores indicating a greater potential to become complacent, or over-trusting, of automation.

Finally, we included a measure to assess patterns of mobility using the Life-space Questionnaire (LSQ; Stalvey et al., 1999). The LSQ is designed to measure the extent and frequency of a person’s mobility in their community. The participants answered how much they travel outside their home, their local community, and their regional area. LSQ is typically administered in an interview format but we adapted it for survey use. Scores could range from 0 (bed-bound) to 9 (could travel out of town daily without assistance).

##### Automation scenarios

Factorial surveys were used to gather subjective assessments of trust in self-driving cars. The survey presented each participant with concrete scenarios of a person interacting with a self-driving car. Factorial surveys are useful when assessing how experimental manipulations affect subjective perceptions, such as trust (Rossi and Anderson, 1982). Additionally, this methodology was used in prior automation research (e.g., Endsley and Kiris, 1995; Mosier and Fischer, 2012; Pak et al., 2014).

In contrast to simple opinion surveys of hypothetical self-driving cars in the extant literature, the current scenarios manipulated the three factors thought to substantially influence driver trust in self-driving vehicles: reliability of the self-driving car (success, failure), risk involved in the scenario (high, low), and physical impairment of the driver in the scenarios (yes, no). Reliability was operationalized as the performance of the self-driving car’s performance. Risk level of the situation was operationalized as the density of traffic or the speed portrayed in the story, with higher density or speed as higher risk because of a higher likelihood of accident. Finally, impairment level of the person was operationalized as the presence of a physical impairment that made driving more difficult. The factorial combinations of the three manipulated factors resulted in 8 unique scenarios. A sample scenario representing a *non-impaired* driver with a *high* reliability car and in a *low risk* situation is represented below:

DJ lives **about 5 miles from the library**. DJ recently bought a car with self-driving capabilities. While sitting in the parking lot of her apartment complex, DJ entered the destination as the library. The car began to drive to the library while she was able to chat on the phone. After a few minutes, DJ **arrived at the library without any issues**.

Key sections that identify the vehicle as reliable (it successfully navigated to destination), the situation as low level of risk (a short trip) are bolded for illustrative purposes. Because no physical impairment is stated or implied, it represented a no-impairment scenario. Below is an example of an *impaired* driver in an *unreliable* car driving in a *high-risk* situation:

JD recently bought a car with self-driving capabilities. She **recently had broken her right foot and was leaving the pharmacy after having picked up some pain medications**. She entered the destination as her home address and the car began to drive. As the car was about to get on **the interstate highway, she noticed that the road was closed with only a small orange road cone**. The car did not know about the new road closure nor could it see the small cone and drove through the road closure. After colliding with some road cones, the car coasted to a safe location and parked itself. Since she could not drive, she had to call her husband.

This scenario represented an unreliable vehicle (it malfunctioned and did not navigate to the destination properly) in a higher risk situation (high speed interstate driving) with a physically impaired driver.

Flesch-Kincaid readability statistics (Kincaid et al., 1975) showed that the mean reading grade level for the scenarios was 7.8. All scenarios were pilot tested to ensure that the factor in the scenarios were noticeable. In the pilot test, younger and older participants read each scenario and judged the reliability of the self-driving car, riskiness of the situation, and impairment of the driver. Pilot participants detected the manipulations in the expected directions.

#### Design and Procedure

The study was a 2 (age group: younger, older adults) × 2 (impairment of driver: yes, no) × 2 (travel risk: lower, higher) × 2 (car reliability: failure, success) mixed-model design with each participant exposed to scenarios that represented combinations of every factor resulting in 8 scenarios. For each scenario, participants were asked to assess their trust on a Likert scale (ranging from 1 to 7). After each scenario, participants were asked the following trust question modeled after Lee and Moray (1994), “To what extent would you trust the self-driving car in this scenario?” Age group, a quasi-independent variable, was the only between-participant manipulation.

Participants were sent a link to complete the experiment. After providing informed consent, they were instructed to complete the experiment in one sitting and to avoid taking breaks. The 8 scenarios were presented in a random order for each participant, one-at-a-time. After judging all 8 scenarios, participants completed CPRS, CPQ-12, and LSQ.

## Results and Discussion

### Demographic Surveys

Technology experience as measured by the computer proficiency questionnaire found that Younger adults scored significantly higher than older adults, *t*(1, 136) = 5.52, *p* < 0.001 (Table 1). Younger adults scored higher in complacency potential than older adults, *t*(1, 136) = 2.64, *p* < 0.01 (Table 1). It is usually more typical to find older adults more complacent than younger adults on the CPRS but the results of previous studies are mixed with some studies finding age differences (Pak et al., 2014) but not others (Pak et al., 2016b). Older adults had a significantly higher life space than younger adults, *t*(1, 136) = -2.84, *p* < 0.01. Older adults were expected to be more sedentary with smaller life space extents (Stalvey et al., 1999), however, our observation may be an artifact of the younger adult sample being drawn from Mechanical Turk; a population who relies on the computer for part of their livelihood, and an older adult sample of persons interested and able to participate in research studies. Participant means are detailed in Table 1.

**Table 1:** Participant characteristics.

	Younger adults *n* = 86			Older adults *n* = 52			Sig. age differences	Cohen’s *d*
				
	Range	*M*	*SD*	Range	*M*	*SD*		
Age	18–51	27.73	5.08	65–87	71.5	5.03		
Technology experience^a^	19–30	28.72	1.94	17.5–30	26.31	3.2	Y > O	0.91
Automation complacency potential^b^	54–80	62.56	5.02	52–73	60.33	4.44	Y > O	0.47
Life space^c^	43,505	5.73	1.26	43,564	6.37	1.28	O > Y	0.5


*^a^Technology experience measured using the Computer Proficiency Questionnaire-short form (Boot et al., 2013). ^*b*^Complacency potential rating scale (Singh et al., 1993). ^*c*^Life space questionnaire (Stalvey et al., 1999).*

### Analysis Approach

The contributions of scenario manipulations (travel risk, car reliability, driver impairment status), individual differences in trust in automation (CPRS), and participant age group on trust of a self-driving car were examined in a two-level hierarchical model. Multiple responses by each participant were nested within the 138 participants where each participant provided a trust rating for 8 scenarios resulting in a total of 1104 analyzable trust judgments. Each judgment was nested within the factorial survey manipulations (high or low travel risk, car successful or not, driver physically impaired). These manipulations were nested within the attributes of the participants (participant age group, participant CPRS score). Multilevel modeling was implemented through SAS, version 9.4 using *proc mixed*.

Analyses via multilevel modeling were chosen due to the hierarchical structure of the nested data. These models account for both within- and between-participant variability as well as cross-level interactions (Raudenbush and Bryk, 2002). Multilevel models account for the non-independence of nested data: the repeated trust assessments made by participants were likely more correlated than responses between participants, violating the assumptions of ANOVA and regression that error variances are independent (Tabachnick and Fidell, 2007). There were also likely to be correlations between different levels (response level, group level). For example, trust responses on a vignette would likely be correlated to the responders age group. Multilevel models prevent the inflated Type I error rate that can occur with use of ANOVA or regression on such nested data. Other literature promotes the use of such models for human factors studies in general (Hoffman and Rovine, 2007). In this study, we used a model building approach where predictors were added in different models and it was noted whether the added predictors improved the fit of the models. The equations for each model are included as an Appendix.

The first model was a fully unconditional (non-multivariate) model (Model 1) that assessed the variance in trust judgments at each level of prediction. This model also provides a baseline to judge the benefit of additional predictors included in other models. Both levels (67% of variance was within-participant, 33% was between participant) contained significant variance, σ^2^ = 2.122, *z* = 21.90, *p* < 0.0001; τ_00_ = 1.03, *z* = 6.54, *p* < 0.0001, allowing for the addition of predictors at each level in the following models.

### Effects of Scenario Manipulations on Trust

Model 2 included main effects of within-participant fixed factors: outcome, risk, impairment, and the interactions of those factors, each of which was significant, and the error term, r_it_, which represents a unique effect associated with the individual (i.e., how much that individual varied across trust judgments). As seen in Table 2, the fixed effects for car reliability outcome (0.43), travel risk (-1.65), and impairment (-0.81) represent the expected linear rate of change in trust judgment for a one-unit increase in those variables. The random effects of within- (σ^2^) and between-individual (τ_00_) variance remained significant at 1.497 (*p* < 0.0001) and 1.107 (*p* < 0.0001), respectively. Supporting our first hypothesis, trust was higher for self-driving cars when it was reliable compared to when the car technology failed, *t*(1, 952) = 2.92, *p* = 0.004. Second, trust was lower when the scenario risk was higher [e.g., high speed expressway driving versus low speed surface roads; *t*(1, 952) = -11.22, *p* < 0.0001]. This result partially did not support our second hypothesis, although the potential moderating effect of age was not explored in this model. Finally, supporting the third hypothesis, the impairment status of the driver influenced trust in the self-driving car: trust was higher when the driver was impaired (e.g., physical impairment that made driving difficult or impossible) compared to when the driver was not impaired, *t*(1, 952) = -5.47, *p* < 0.0001.

**Table 2:** Multilevel modeling results.

	Model 1		Model 2		Model 3	
			
	Unconditional		Between-person manipulations		Within- and between-person manipulations	
**Fixed Effects**						
	**Estimate**	**SE**	**Estimate**	**SE**	**Estimate**	**SE**

Intercept	4.583^***^	0.097	4.870^***^	0.137	4.726^***^	0.168
**Between-person**						
AgeGroup, γ_01_					0.38	0.269
CPRS, γ_02_					0.074^***^	0.019
**Within-person**						
Outcome, γ_10_			0.432^*^	0.148	0.650^***^	0.178
Risk, γ_20_			-1.652^***^	0.147	-1.531^***^	0.177
Impairment, γ_30_			-0.810^***^	0.148	-0.872^***^	0.178
Outcome^∗^Risk, γ_40_			1.496^***^	0.209	1.357^***^	0.237
Outcome^∗^Impairment, γ_50_			0.764^***^	0.21	0.892^***^	0.239
Risk^∗^Impairment, γ_60_			1.346^***^	0.209	1.403^***^	0.237
Outcome^∗^Risk^∗^Impairment, γ_70_			1.365^***^	0.296	-1.372^***^	0.294
**Cross-level interactions**						
Agegroup^∗^Outcome, γ_11_					-0.580^*^	0.262
Agegroup^∗^Risk, γ_21_					-0.322	0.262
Agegroup^∗^Impairment, γ_31_					0.154	0.262
Agegroup^∗^Outcome^∗^Risk, γ_41_					0.367	0.303
Agegroup^∗^Outcome^∗^Impairment, γ_51_					-0.321	0.303
Agegroup^∗^Risk^∗^Impairment, γ_61_					-0.14	0.303
**Random Effects**						
σ^2^	2.122^***^	0.097	1.497^***^	0.069	1.478^***^	0.068
τ_00_	1.025^***^	0.157	1.107^***^	0.157	0.990^***^	0.144
**Model Fit Statistic**						
A1C	4162		3837.5		3819.2	


*^∗^Means significant at *p* < 0.05; ^∗∗∗^ means significant at *p* < 0.001.*

**FIGURE 1:**
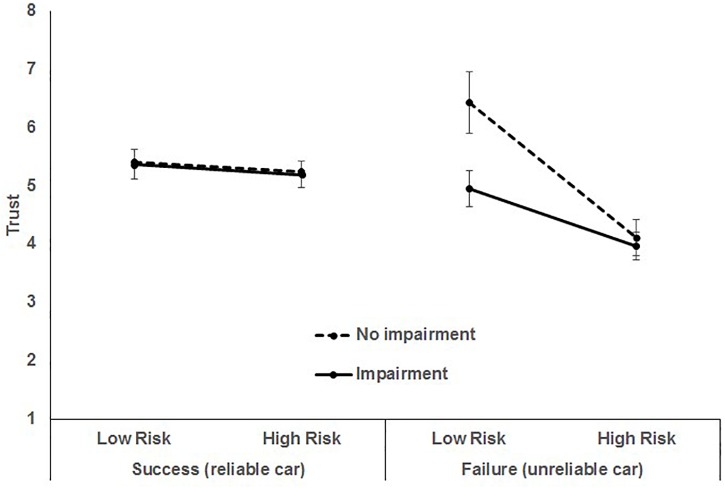
3-way interaction of car reliability, risk, and impairment. Bars represent 95% CI.

Hypothesis 4, predicting an interaction of the three situational factors (travel risk, reliability, driver impairment) to affect trust in the self-driving car, was also supported, *F*(1, 952) = 21.34, *p* < 0.0001 (Figure 1).

**FIGURE 2:**
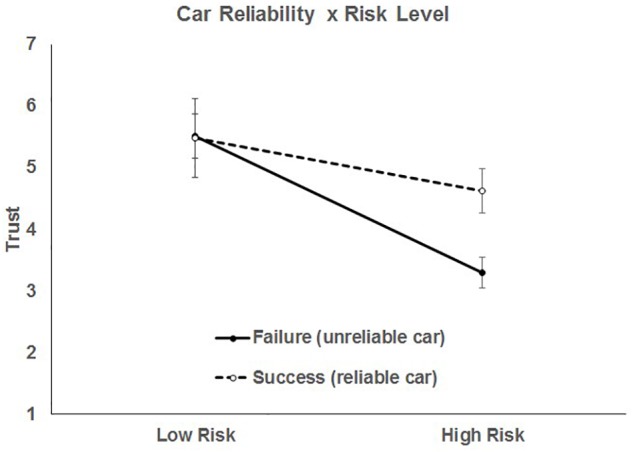
Two-way interaction between car reliability and risk level. Bars represent 95% CI.

The three way interaction can be explained by the presence of several significant two-way interactions. First, the two-way interaction of car reliability and risk was significant, *F*(1, 952) = 51.36, *p* < 0.0001, indicating that the effect of reliability on trust varied as a function of risk, illustrated in Figure 2.

For the car reliability manipulation, the slopes of both failure, *t*(946) = -10.3, *p* < 0.0001, and success, *t*(946) = -8.66, *p* < 0.0001, were significantly different from zero. No matter the car reliability, people reported lower trust in high risk situations. The interaction comes from the differences in trust for car failures and successes for different levels of travel risk. When a scenario was low risk, car reliability had no effect on trust, *t*(946) = 1.35, = 0.176. However, in high risk scenarios, people reported lower trust in the car when it failed than when it succeeded, *t*(946) = 11.3, *p* < 0.0001.

**FIGURE 3:**
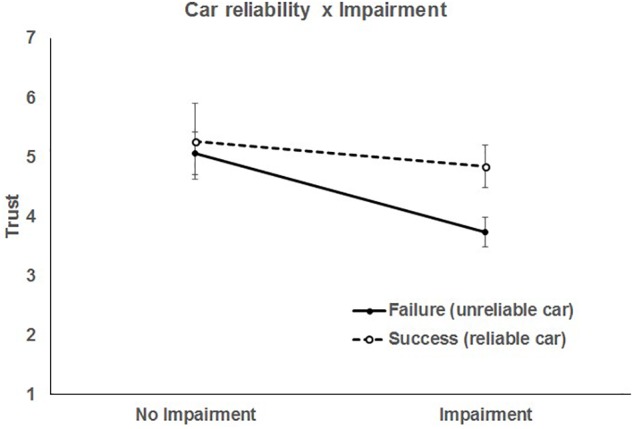
Two-way interaction between car reliability and driver impairment. Bars represent 95% CI.

The second significant 2-way interaction was between car reliability and driver impairment, *F*(1, 952) = 13.24, *p* = 0.0003, indicating that trust in the automation due to car reliability varied as a function of the impairment status of the driver (Figure 3).

There was no difference in trust between when the car technology failed or succeeded when the driver had no impairment, *t*(946) = 0.21, *p* = 0.832, but trust was lower when the car technology failed compared to when it succeeded only when the driver had an impairment, *t*(946) = 7.75, *p* < 0.0001. That is, when the driver was not seen to be impaired, the self-driving car’s performance did not affect trust. However, when the driver was thought to be impaired, respondents had less trust when the car technology failed. This was indicated by slopes significantly different from zero for both failure, *t*(946) = -5.09, *p* < 0.0001, and success, *t*(946) = -4.08, *p* > 0.0001. We speculate this was due to a belief that an impaired driver could not compensate for the failure of self-driving automation. It is also inconsistent with the notion that when the potential benefits of automation are made evident, adoption and trust may be enhanced.

**FIGURE 4:**
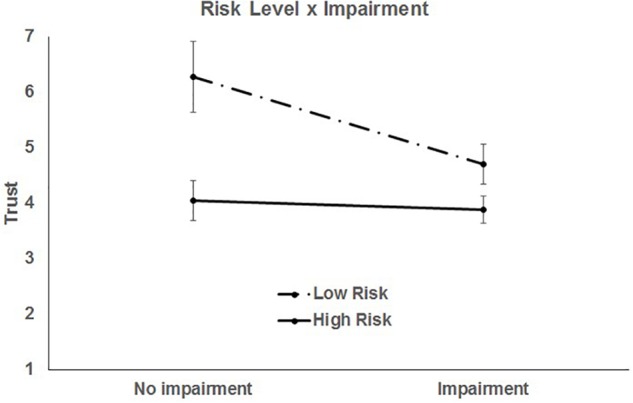
Two-way interaction of risk and impairment. Bars represent 95% CI.

The final significant two-way interaction was between travel risk and impairment level of the driver, *F*(1, 952) = 41.56, *p* < 0.0001 (Figure 4).

Trust was lower for scenarios with impaired drivers, but this was qualified by risk level. Trust was higher in general for low risk scenarios, both when the driver was impaired, *t*(946) = -9.38, *p* < 0.0001, and not impaired, *t*(946) = -9.98, *p* < 0.0001. In high risk scenarios, there was no difference in trust by impairment, *t*(946) = -1.31, *p* = 0.191. That is, when the driving risk was perceived to be high, trust in the self-driving car was unaffected by the driver’s impairment status (trust was already relatively low). However, when the travel risk was lower, trust in the self-driving car was less for an impaired driver compared to a non-impaired driver, *t*(946) = -6.34, *p* < 0.0001. This might indicate a latent mistrust in the ability of the automation to handle driving situations when the driver is impaired. It is also, again, inconsistent with the notion that when scenarios are presented where the automation may prove useful (i.e., the potential benefits are clearly stated), adoption and trust might be enhanced.

To summarize the effects on trust in the car: when the car was reliable, trust in the automation was high across all levels of risk and impairment. Said another way, riskiness of the situation or driver status had little to no effect on trust when the car was reliable. However, when the car performed poorly (failure), trust was negatively impacted by risk and driver status such that with impaired drivers, trust declined only slightly with increased risk. However, when the driver was impaired, trust in the car significantly declined with increased risk. Twenty nine percent of the within-person variance in trust was accounted for by the scenario manipulations. This interaction of car reliability, travel risk, and driver impairment status suggested that trust, and ultimately acceptability, adoption, and usage of self-driving cars, is not only dynamic but highly specific and more nuanced than previously thought (Schoettle and Sivak, 2015, 2016).

### Effects of Scenario Manipulations and Age on Trust

Model 3 contained the within-participant (Level 1) predictors of Model 2, the addition of age group as a between-participant (Level 2) variable, and hypothesized cross-level interactions. CPRS Score was examined as a main effect and controlled for in the examination of the interactions. Within-participant effects maintained their direction and significance (Table 2). The random effects of within- (σ^2^) and between-individual (τ_00_ ) variance remained significant at 1.478 (*p* < 0.0001) and 0.990 (*p* < 0.0001), respectively.

**FIGURE 5:**
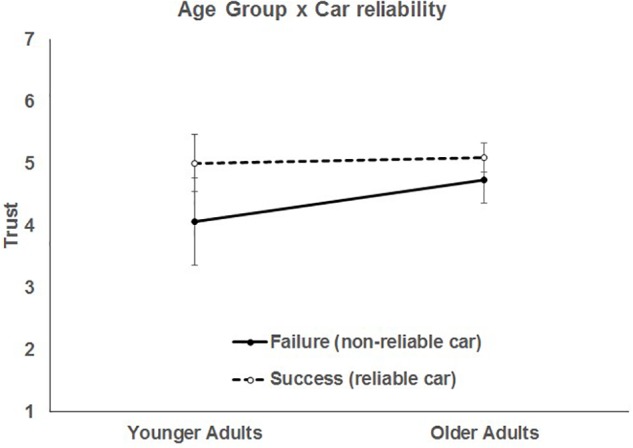
Two-way interaction of age group and car reliability. Bars represent 95% CI.

There was no main effect of age group, *F*(1, 135) = 1.99, *p* = 0.160. People with high CPRS scores tended to report higher trust in the car, *t*(1, 946) = 3.84, *p* = 0.0002. Although there were no significant differences between the average trust ratings of younger and older adults, there was a significant interaction of age group by car reliability, *F*(1, 946) = 4.89, *p* = 0.027), such that younger adults adjusted their trust downward for automation failures more than older adults (Figure 5). The responsiveness of younger adult’s trust to the automation reliability in contrast to older adults might be an indication of older adults’ increased complacency with automation and is consistent with prior age-related automation studies (e.g., Pak et al., 2016b).

More reliable cars led to higher trust for both younger, *t*(946) = 3.44, *p* = 0.0006, and older adults, *t*(946) = 2.4, *p* = 0.017. Although the interaction of age group and car reliability was significant, neither the slope for automation failure, *t*(946) = 1.9, *p* = 0.057, or success, *t*(946) = 0.38, *p* = 0.702, was significantly different than 0. The significant 2 and 3-way interactions from Model 2 retained their direction and significance even when age group and CPRS were controlled for. This model accounted for 3% of the between person variance and 30% of the within-person variance in trust. Contrary to Hypothesis 2, we did not find that trust differed for younger and older adults depending on travel risk – respondents had similar trust reactions to the manipulations of risk in the scenarios.

## Conclusion

Just as older adults are disproportionately harmed in highway accidents they may reap the most benefits with the adoption of self-driving technology. Self-driving cars could dramatically increase the number of annual vehicle miles traveled for older adults (Harper et al., 2016). In addition, older adults’ travel patterns indicate their strong preference for the use of personal vehicles over alternative solutions and that their trips tend to be of shorter length and duration (Collia et al., 2003). This makes older adults who are at risk for driving cessation ideal candidates for self-driving vehicles because it addresses the major problem of personal mobility but minimizes the risk of older adults continuing to drive with age related impairments.

However, great caution is still warranted as the simple introduction of a high level of automation may cause unanticipated issues. It is a very common misconception that machines (automation) are always more accurate and capable than humans and that safety and efficiency will be enhanced by replacing humans with automation (for the latest refutation of this misconception in the context of medical errors see Semigran et al., 2016). This view also ignores the continuous work of Parasuraman and colleagues (Parasuraman and Riley, 1997; Parasuraman, 2000; Parasuraman and Wickens, 2008; Parasuraman and Manzey, 2010) that has clearly demonstrated the complex and sometimes counter-intuitive human performance consequences of interacting with highly reliable but ultimately imperfect automation.

The primary contribution of this work is that the ratings of trust in self-driving cars varied with situational characteristics (reliability, driver impairment, risk level). These results also stand in contrast to past cross-sectional opinion surveys of drivers that showed little change in the negative perception of self-driving cars over time (Schoettle and Sivak, 2015, 2016). Our findings were consistent with the notion that when drivers are provided with additional information, their perceptions of driving related technologies adjusts accordingly (Biassoni et al., 2016). Another interesting finding was that in contrast to extant opinion surveys of self-driving vehicles, there were few age differences in trust. This was surprising as there are well-known age differences in technology experience and attitudes (Czaja et al., 2006; Van Volkom et al., 2014). It was also surprising because there are well-documented age differences in attitudes and behavior toward automation (Mouloua et al., 2002).

Additional research should examine how to enhance the process of trust recovery with self-driving cars after an inevitable malfunction. Instead of relying on the passage of time for trust recovery, more active processes may be used to make sure that trust recovers quickly after a failure. Not addressing trust recovery may cause older drivers to abandon otherwise reliable automation and assume more dangerous manual control. The dynamics of trust recovery have been extensively examined in human-human interactions (e.g., Dirks et al., 2011) and recently explored in human-automation interactions (de Visser et al., 2018). This is a key area for further research because older adults have been shown to have different time course of trust recovery compared to younger adults (Sanchez et al., 2014).

One unexamined issue with self-driving vehicles is the issue of locus of control. Locus of control is a context-dependent individual difference in the amount of control one believes one has in a situation (Rotter, 1966). Locus of control and perceived control is contained within many behavior models, such as the Theory of Planned Behavior (Ajzen, 1985) and the Integrated Behavioral Model (Montaño and Kaspryzk, 2008) to predict intentions and finally, behavior. By its definition, self-driving vehicles assume full control from the driver–this full control may interact with the locus of control beliefs of the driver to affect their behavior. Stanton and Young (2000) discussed the possible unexplored issue of the decrease in internal control beliefs with highly automated driver assistance systems such as automated cruise control especially because of the existence of age-related differences in locus of control (Lachman, 1986). We predict these findings would extend to riders of self-driving vehicles and also their attributions of the behavior with self-driving vehicles and extant age differences in locus of control might explain any age differences in trust.

Finally, related to the notion of locus of control is the issue of individual differences with automation. Currently, many driver assistance systems, including self-driving cars, are implemented and designed without regard for individual differences–they are simply offered on cars with no room for customizability. However, it is well known that individual differences in personality (Parasuraman et al., 1993) and cognitive abilities (e.g., Pak et al., 2016a; Rovira et al., 2016) can influence not only how one performs with automation, but how they perceive it (trust). Individual differences are also expected to play a greater role in explaining older adults’ behavior with automation simply because aging is associated with greater variability in individual differences (Morse, 1993; Hultsch et al., 2000, 2002).

### Limitations

The main limitation of this work is the lack of use of a real world autonomous car. This limits participants’ responses to be based on notions of self-driving vehicles versus actual experience. Additionally, the authors did not fully account for the media impact of accidents due to self-driving cars on trust individuals’ trust ratings.

Another limitation is that younger adults recruited were more sedentary than older adult participants based on their responses to the Life Space Questionnaire. As noted earlier, the lower Life Space Questionnaire scores observed from the young population may be an artifact of the younger adult sample being drawn from Mechanical Turk; a population who relies on the computer for part of their livelihood. Literature has shown that data from Mturk is as reliable as data obtained from traditional methods (Buhrmester et al., 2011). Additionally, our older adult sample is drawn from individuals interested and able to participate in research studies. Lastly, the Life Space Questionnaire is predominantly used with older adult populations to demonstrate they aren’t sedentary. There is very little data with the use of the survey with young populations, hence it is possible that the lower Life Space scores may be a result of a variety of factors including being on college campuses, not having a car, or the increase in technology enabling individuals to stay connected without having to leave their vicinity often.

### Design Guidelines for Self-Driving Vehicles

To support appropriate trust calibration and driver engagement self-driving cars should:

(1) Alert the driver that a high risk situation would arise based on the projected route.
(2) Alert the driver that a potentially uncertain situation (potential failure) is eminent.
(3) If a high risk situation would arise based on the projected route do not allow an impaired driver to execute the high risk route.
(4) Given the finding that older adults do not downwardly adjust their trust as much as young adults, when a potentially uncertain situation (potential failure) is eminent, provide a longer lead time for older adults to assess and re engage.

In summary, the guidelines revolve around solutions for how to support driver recovery from performance decrements as a result of out of the loop unfamiliarity related to imperfect automation or high risk situations. Where out of the loop familiarity behavior refers to an operators expectation that the automation will safely control a system and thus is caught not attending to events in the environment and finds it difficult to re engage and provide corrective actions in the event of imperfect automation (Wickens and Hollands, 2000). The guidelines would specifically force individuals to periodically re-enter the loop, ensure designers resist the temptation to impose high levels of automation with safety critical operations, and making what the automation is doing transparent (Chen et al., 2014; Wickens, 2019, personal communication, February 12, 2019).

## Ethics Statement

This study was carried out in accordance with the recommendations of “Clemson University’s IRB” with written informed consent from all subjects. All subjects gave written informed consent in accordance with the Declaration of Helsinki. The protocol was approved by the “Clemson University’s IRB.”

## Author Contributions

ER, RP, and AM contributed to the conception and design of the study. AM performed the statistical analysis. ER, RP, AM, and LH wrote sections of the manuscript. All authors contributed to manuscript revision, read and approved the submitted version.

## Conflict of Interest Statement

The authors declare that the research was conducted in the absence of any commercial or financial relationships that could be construed as a potential conflict of interest.

## Appendix

**Equation for Model 1**

Level 1: Trust_it_ = β_0it_ + r_it_

Level 2: β_0i_ = γ_00_ + u_0i_

**Equation for Model 2**

$$\begin{array}{cc} \mathrm{Level}\;1:\;{\mathrm{Trust}}_{\mathrm{it}}\;= & \beta_{0\mathrm{it}}+\beta_{1\mathrm{it}}(\mathrm{Outcome})+\beta_{2\mathrm{it}}(\mathrm{Risk})+\beta_{3\mathrm{it}}(\mathrm{Impairment})+\beta_{4\mathrm{it}}(\mathrm{Outcome}*\mathrm{Risk})+\\ & \beta_{5\mathrm{it}}(\mathrm{Risk}*\mathrm{Impairment})+\beta_{6\mathrm{it}}(\mathrm{Impairment}*\mathrm{Outcome})+\\ & \beta_{7\mathrm{it}}(\mathrm{Risk}*\mathrm{Impairment}*\mathrm{Outcome})+r_{\mathrm{it}} \end{array}$$

$$\begin{array}{ll} \mathrm{Level}\;2: & \beta_{0i}=\gamma_{00}+u_{0i}\\ & \beta 1i=\mathrm{\gamma 10}\\ & \beta 2i=\mathrm{\gamma 20}\\ & \beta 3i=\mathrm{\gamma 30}\\ & \beta 4i=\mathrm{\gamma 40}\\ & \beta 5i=\mathrm{\gamma 50}\\ & \beta 6i=\mathrm{\gamma 60}\\ & \beta 7i=\mathrm{\gamma 70} \end{array}$$

**Equation for Model 3**

$$\begin{array}{cc} \mathrm{Level}\;1:\;{\mathrm{Trust}}_{\mathrm{it}}\;= & \beta_{0\mathrm{it}}+\beta_{1\mathrm{it}}(\mathrm{Outcome})+\beta_{2\mathrm{it}}(\mathrm{Risk})+\beta_{3\mathrm{it}}(\mathrm{Impairment})+\beta_{4\mathrm{it}}(\mathrm{Outcome}*\mathrm{Risk})+\\ & \beta_{5\mathrm{it}}(\mathrm{Risk}*\mathrm{Impairment})+\beta_{6\mathrm{it}}(\mathrm{Impairment}*\mathrm{Outcome})+\\ & \beta_{7\mathrm{it}}(\mathrm{Risk}*\mathrm{Impairment}*\mathrm{Outcome})+r_{\mathrm{it}} \end{array}$$

$$\begin{array}{ll} \mathrm{Level}\;2: & \beta_{0i}=\gamma_{00}+\gamma_{01}(\mathrm{AGEGROUP})+\gamma_{02}(\mathrm{CPRS})+u_{0i}\\ & \beta_{1i}=\gamma_{10}+\gamma_{11}(\mathrm{AGEGROUP})\\ & \beta_{2i}=\gamma_{20}+\gamma_{21}(\mathrm{AGEGROUP})\\ & \beta_{3i}=\gamma_{30}+\gamma_{31}(\mathrm{AGEGROUP})\\ & \beta_{4i}=\gamma_{40}+\gamma_{41}(\mathrm{AGEGROUP})\\ & \beta_{5i}=\gamma_{50}+\gamma_{51}(\mathrm{AGEGROUP})\\ & \beta_{6i}=\gamma_{60}+\gamma_{61}(\mathrm{AGEGROUP})\\ & \beta_{7i}=\gamma_{70} \end{array}$$
